# Impact of Excipients and Seeding on the Solid-State Form Transformation of Indomethacin during Liquid Antisolvent Precipitation

**DOI:** 10.1021/acs.cgd.2c00678

**Published:** 2022-09-09

**Authors:** Mariana Hugo Silva, Ajay Kumar, Benjamin K. Hodnett, Lidia Tajber, René Holm, Sarah P. Hudson

**Affiliations:** † Pharmaceutical Product Development and Supply, Janssen Research and Development, Division of Janssen Pharmaceutica NV, Turnhoutseweg 30, 2340 Beerse, Belgium; ‡ Department of Chemical Sciences, SSPC the Science Foundation Ireland Research Centre for Pharmaceuticals, Bernal Institute, University of Limerick, Castletroy, Co., Limerick V94 T9PX, Ireland; § School of Pharmacy and Pharmaceutical Sciences and the Science Foundation Ireland Research Centre for Pharmaceuticals (SSPC), Trinity College Dublin, College Green, Dublin 2 D02 PN40, Ireland; ∥ Department of Physics, Chemistry and Pharmacy, University of Southern Denmark, Campusvej 55, 5230 Odense, Denmark

## Abstract

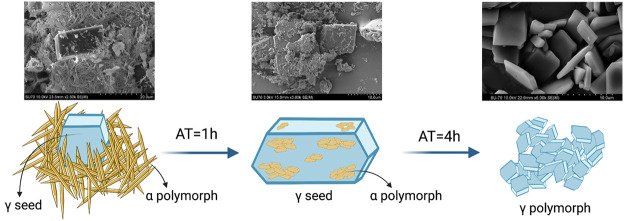

Long-acting injectables are a unique drug formulation strategy, providing a slow and sustained release of active pharmaceutical ingredients (APIs). In this study, a novel approach that combines liquid antisolvent precipitation with seeding to obtain a stable form of the API indomethacin while achieving the desired particle size distribution is described. It was proven that when a metastable form of indomethacin was initially nucleated, the rate of its transformation to the stable form was influenced by the presence of excipients and seeds (17.10 ± 0.20 μm), decreasing from 48 to 4 h. The final particle size (D50) of the indomethacin suspension produced without seeding was 7.33 ± 0.38 μm, and with seeding, it was 5.61 ± 0.14 μm. Additionally, it was shown that the particle size distribution of the seeds and the time point of seed addition were critical to obtain the desired solid-state form and that excipients played a crucial role during nucleation and polymorphic transformation. This alternative, energy-efficient bottom-up method for the production of drug suspensions with a reduced risk of contamination from milling equipment and fewer processing steps may prove to be comparable in terms of stability and particle size distribution to current industrially accepted top-down approaches.

## Abstract 2

A novel liquid antisolvent technology to generate suspensions of active pharmaceutical ingredients with a target particle size distribution and a polymorphic form was developed. Using indomethacin as a model, this technology, combined with seeding, drives and accelerates the solid-state form transformation from a metastable form to a stable polymorphic form. The seeds act as a template for the metastable form transformation.

## 1 Introduction

Long-acting injectables (LAIs) are a unique drug formulation strategy, providing a slow and sustained release of active pharmaceutical ingredients (APIs) for weeks to months after administration.^1^ LAI formulations present several advantages over traditional oral formulations, including effective drug usage, reduced frequency of administration, enhanced therapy adherence (i.e., compliance), and mitigation of possible adverse effects by avoiding peak plasma concentrations. Together, these elements can lead to an improved quality of life for patients.^2−7^ LAIs include a very broad range of dosage forms, e.g., encapsulated systems such as drug-loaded micro-/nanospheres, micro-/nanocapsules, and solid drug particle systems.^8^ Aqueous suspensions containing solid drug particles can be produced in micro-/nanosize ranges and often have a stabilizer or surfactant shield to stabilize the particle size distribution and particle morphology during storage. These injectable systems can be lyophilized into dry powders (for reconstitution before administration) or preferably be maintained as liquid suspensions ready for injection.^9^ More recently, the popularity of LAIs consisting of aqueous suspensions of solid drug particles, where the low solubility and slow dissolution rate of the solid itself control the rate of release, has re-emerged.^10,11^ Crystalline aqueous solid drug suspensions are a well-established drug delivery platform, with several drug products already approved for marketing and even more under development for different therapeutic areas. In these formulations, a particle size distribution between 5 and 10 μm is usually applied.^12^ Although research in this field has increased in recent years, methods to manipulate the particle size (micro-/nanoparticles) and morphology require further development, as these are critical product quality attributes for the systems.

There are two methodologies for producing solid micro-/nanoparticles: (1) a top-down approach (i.e., diminution of the particles to achieve the required size) or (2) a bottom-up approach (i.e., precipitation of the drug from a solution). The production method will influence particle characteristics, such as particle size, crystallinity, and surface properties, as well as selection of stabilizers. Therefore, the chosen method of production may play a critical role in drug suspension stability, composition, and subsequent release profile upon administration. Stabilizing agents can be added in low concentrations during or after production to achieve the desired particle size distribution, prevent subsequent particle growth and irreversible particle agglomeration, and improve resuspendability before injection.^13^ Currently, the selection of stabilizers for suspensions is based on a trial-and-error approach for both top-down and bottom-up approaches. However, to the best of our knowledge, a correlation in stabilizer selection for the two approaches has never been investigated.^14−18^

All current marketed LAI products are manufactured using top-down methods. In this approach, larger drug particles or crystals are typically suspended in a dispersion medium and mechanically reduced into micro-/nanoparticles, but dry milling approaches can also be applied. The milling is driven by cavitation forces, shear forces, and collisions between particles.^19,20^ The most commonly used top-down techniques are wet and jet milling, homogenization using a rotor and a stator or high-pressure homogenization, and microfluidization.^19,21^ The particles produced by these top-down approaches must fit the requirements for industrial scale-up. Particles need to have the required brittleness to obtain the desired particle size, but it can be difficult to obtain a uniform size distribution of particles in the case of hard materials.^22^ Furthermore, the top-down methods are in general time-consuming, requiring a significant amount of energy and increasing the chance of amorphization. The number of cycles required depends on the hardness of the drug, and due to the mechanical nature of this process, contamination from the media milling or from the homogenization chamber can occur.^13,23−25^

Bottom-up methods produce fine particles from solution, building up from the molecular level. This can enable more versatility for the particle properties (e.g., size, morphology, surface properties, and crystallinity) when compared to top-down approaches.^26^ There are different ways to crystallize drugs from solution, and some examples are given in Table 1. Liquid antisolvent (LAS) precipitation is one of the most attractive bottom-up techniques. This approach involves dissolution of a poorly water-soluble drug in a solvent (in which it is highly soluble), which is then mixed with a miscible antisolvent (in which the drug is poorly soluble), generating a high supersaturation level, which results in fast nucleation, leading to the production of micro-/nanoparticles. The precipitated particles tend to grow bigger in size over time due to the Ostwald ripening phenomenon. Therefore, stabilization of aqueous suspensions of particles is critical for long-term storage.^27^ Thorat et al. reviewed more than 50 cases of LAS precipitation and reported that some of the excipients used in these formulations were common to existing commercial LAIs produced by the top-down method, e.g., poloxamer, hydroxypropyl methyl cellulose (HPMC), poly(vinylpyrrolidone) (PVP), poly(ethylene glycol) (PEG), etc.^27^ To prevent particle growth in suspensions, isolation to dryness is frequently necessary and is achieved by filtering, freeze-drying, or spray-drying.^28^ LAS precipitation is also easily scalable.^27^ The simplicity of this technique, the shorter processing time, fewer process steps, independence from compound brittleness, and low-cost equipment make it easily scalable and hence relevant and of interest to the pharmaceutical industry. However, particle growth by agglomeration or Ostwald ripening can be difficult to control and needs further exploration. A selection of excipients used to stabilize API drug particles produced by bottom-up approaches are presented in Table 1. The examples were selected because the excipients used are common to commercially available LAIs.

**Table 1:** Selection of Excipients Used in Bottom-Up Approaches for Production of API Drug Particles^a^

method	excipient	API	reference
cooling crystallization	HPMC	paracetamol	(29)
LAS precipitation	poloxamer 407, HPMC E5	bicalutamide	(30)
LAS precipitation	poloxamer 188	taxifolin	(31)
LAS precipitation	PEG 4000	ascorbyl palmitate α	(27)
LAS precipitation	PVP K30	felodipine	(27)
LAS precipitation	Tween 80	sirolimus	(27)
LAS precipitation	stearic acid	theophylline	(27)
evaporative precipitation into aqueous solution	PVP K15, poloxamer 407	carbamazepine	(32)
evaporative precipitation into aqueous solution	Tween 80, PVP K15, PEG 40,000, PEG 8,000	cyclosporine A	(33)
spray drying	PVP K30	curcumin	(34)
spray drying	Tween 80	bovine serum albumin	(35)
SAS	PVP	curcumin, ezetimibe, etc.	(36)
SAS	PEG	itraconazole, carotene, etc.	(36)
SAS	ethyl cellulose	ampicillin, amoxicillin, etc.	(36)
ASES	HPMC	itraconazole	(37)
GAS	ethyl cellulose	carbamazepine	(38)

a All excipients have also been used in commercially available LAIs. LAS, liquid antisolvent; SAS, supercritical antisolvent; GAS, gas antisolvent; ASES, aerosol solvent extraction system.

In this work, indomethacin was used as a model drug. The molecular formula of indomethacin, also known as 2-[1-(4-chlorobenzoyl)-5-methoxy-2-methylindol-3-yl]acetic acid, is C_19_H_16_ClNO_4_, with a molecular weight of 357.79 g/mol.^39^ Comer et al. determined the intrinsic solubility of indomethacin in water and obtained the value of 8.8 μg/mL at 25 °C for the γ form.^40^ Indomethacin is considered a BCS class II drug (i.e., high permeability and low solubility).^41^ Indomethacin is freely soluble in organic solvents, such as acetone and ethyl acetate, and sparingly soluble in ethanol or methanol.^42^ Indomethacin has seven reported polymorphic forms: α, β, γ, δ, ε, ζ, and η, with the α and γ forms being the most commonly obtained. The γ form of indomethacin is the thermodynamically stable form of the currently known forms, and α is the most common metastable form obtained when crystallizing indomethacin.^43^

Seeding has been used in this project as a technique for expediting the solid-state transformation of a metastable form to a stable form, without negatively impacting the resulting particle size distribution, controlled by the LAS process parameters. Table 2 summarizes previous applications of seeding during bottom-up approaches in the literature, detailing the purpose of the seeding and the nucleation mechanism involved.

**Table 2:** Literature Review of the Application of Seeding during Bottom-Up Approaches

API	technique	purpose of seeding	type of nucleation	ref
fesoterodine fumarate	cooling crystallization	tailor particle size distribution	secondary nucleation	(44)
paracetamol	cooling crystallization and milling	improve crystal size and/or shape	primary homogeneous and secondary nucleation	(45)
l-glutamic acid	oscillatory baffled crystallizer	phase transformation study	secondary nucleation	(46)
indomethacin	antisolvent crystallization	increase crystallization kinetics	secondary nucleation	(47)
paracetamol	cooling crystallization	control over supersaturation—nucleation (impact on metastable zone width)	primary homogeneous	(48)
glycine	cooling crystallization	tailor particle size distribution	secondary nucleation	(49)
ammonium sulfate	cooling crystallization	tailor particle size distribution	secondary nucleation	(50)
paracetamol	antisolvent crystallization	tailor particle size distribution	primary homogeneous	(51)
sulfathiazole	cooling crystallization	control of size uniformity and polymorphic purity	primary homogeneous	(52)
hydroxytriendione	cooling crystallization	polymorphic purity	secondary nucleation	(53)
abecarnil	cooling crystallization	control crystallized polymorph	secondary nucleation	(54)
treprostinil diethanolamine	cooling crystallization	control crystallized polymorph	primary homogeneous	(55)
potassium dichromate	cooling crystallization	tailor particle size distribution	secondary nucleation	(56)
glycine	cooling crystallization	control size uniformity and polymorphic form	secondary nucleation	(57)
lovastatin	impinging jet crystallization	tailor particle size distribution	secondary nucleation	(58)
magnesium sulfate	stirred-tank crystallizers	influence on the nucleation rate	secondary nucleation	(59)
glycine	cooling crystallization	control crystallized polymorph	secondary nucleation	(60)

As shown in Table 2, Malwade and Qu previously crystallized indomethacin via the use of seeds in a liquid antisolvent precipitation process.^47^ In their experiments, the nucleation of the α form was observed with subsequent transformation to the γ form when seeds of the γ form were added.^47^ However, there was no attempt to control the final particle size distribution in their report, a critical quality attribute for the development of LAI suspensions. Thus, the generation of an LAI suspension of the stable form of indomethacin, polymorph γ, with a target particle size distribution (PSD, 5–10 μm) via an LAS precipitation method has not yet been reported. While LAI formulations of indomethacin would not be of interest therapeutically, its challenging polymorphic behavior makes it an interesting model system for designing an LAS precipitation method that would result in a stable LAI suspension with a target particle size distribution and the desired polymorphic form. Critical parameters during the LAS precipitation to achieve the target particle size and polymorphic form (e.g., temperature, aging time, need for seeding, antisolvent/solvent ratio, stirring rate, and excipient selection) were evaluated.

## 2 Materials and Methods

### 2.1 Materials

Indomethacin was purchased from Acros Organics (Fisher Scientific, Geel, Belgium). Hydroxypropylmethylcellulose 2910, 5 mPa s (HPMC E5) was purchased from DDP Specialty Electronic Materials (DDP Speciality Electronic Materials Plaquemine). Poloxamer 407, poly(vinylpyrrolidone) (PVP) K30, and sodium lauryl sulfate (SLS) were obtained from BASF (BASF Chemtrade GmbH, Burghernheim, Germany). Docusate sodium salt (DOSS) was purchased from Merck KGaA (Darmstadt, Germany). Purified water was freshly prepared using a Milli-Q integral water purification system (Milli-Q Advantage A10; Merck Millipore, Merck A/S, Hellerup, Denmark). Ethanol for analysis was obtained from Merck KGaA (Merck KGaA, Darmstadt, Germany). Acetone, laboratory reagent grade ≥99%, was purchased from Fisher Scientific (Fisher Scientific, Loughborough, U.K.). All suspensions were filtered with a Durapore 0.22 μm PVDF Membrane (Merck Millipore Ltd., Cork, Ireland) prior to analysis by X-ray powder diffraction (PXRD) and scanning electron microscopy (SEM).

### 2.2 Methods

#### 2.2.1 Preparation of Indomethacin Suspensions by LAS Precipitation—without Seeding

##### 2.2.1.1 Optimization of LAS Precipitation Process Parameters

During LAS precipitation, 1 mL of indomethacin solution in ethanol was quickly introduced into water. The antisolvent process conditions were optimized by monitoring the effects of drug concentration (9, 10, 25, 50 mg/mL), solvent-to-antisolvent (S/AS) volume ratio (1:20, 1:10), agitation rate (500 or 1200 rpm), and aging time (up to 60 min) on the resulting PSD and morphology after precipitation. The LAS precipitation experiments were conducted at 5 and 25 °C using a temperature-controlled water bath Grant TFX200 (Grant Instruments Ltd., Shepreth, U.K.).

##### 2.2.1.2 Effect of Excipients on the Antisolvent Process

During LAS precipitation, 1 mL of indomethacin solution in ethanol (10 or 15 mg/mL) was quickly introduced into water (10 mL) with or without excipients at different compositions (see Table 3). Excipients were either dissolved in the antisolvent and present during nucleation or dissolved in water and added to the S/AS mixture after nucleation. Solutions/suspensions were maintained at a constant temperature of 5 or 25 °C under rapid agitation at 1200 rpm (Spinbar disposable magnetic stirring bar) throughout the precipitation process. On contact with the antisolvent, indomethacin immediately precipitated, giving a milky suspension.

**Table 3:** Summary of LAS Precipitation Process Parameters Tested at 25 °C with an S/AS Ratio of 1:10, a Stirring Rate of 1200 rpm, and Ethanol as a Solvent

formulation	API concentration (mg/mL)	excipients	time of addition
1	10	poloxamer 407 1% w/v	excipient present at the time of addition of the drug solution
2	10	PVP K30 1% w/v	
3	10	DOSS 0.1% w/v	
4	10	HPMC 0.1% w/v	
5	10	DOSS 0.1% w/v + PVP K30 1% w/v	surfactant present at the time of addition of the drug solution, polymer solution added 10 s later
6	10		both surfactant and polymer present at the time of addition of the drug solution
7	10		surfactant present at the time of addition of the drug solution, polymer solution added 20 s later
8	10		surfactant present at the time of addition of the drug solution, polymer solution added 30 s later
9	15		surfactant present at the time of addition of the drug solution, polymer solution added immediately after
10	10	PVP K30 1% w/v	polymer added 10 s later after the drug solution.
11	10		polymer added 20 s later after the drug solution
12	10	DOSS 0.1% w/v + poloxamer 407 1% w/v	surfactant present at the time of addition of the drug solution, polymer solution added 10 s later
13	15		surfactant present at the time of addition of the drug solution, polymer solution added immediately after
14	10		surfactant and polymer added 10 s later
15	10	SLS 1% w/v + poloxamer 407 1% w/v	surfactant present at the time of addition of the drug solution, polymer solution added 10 s later
16	10		surfactant present at the time of addition of the drug solution, polymer solution added immediately after
17	10		surfactant and polymer added 10 s after addition of the drug solution

#### 2.2.2 Preparation of Indomethacin Suspensions by Liquid Antisolvent Precipitation—with Seeding

##### 2.2.2.1 Production of Indomethacin γ Seeds

Seeds of the γ polymorphic form were obtained by LAS precipitation by quickly injecting 1 mL of indomethacin solution in ethanol (10 mg/mL) into water (10 mL) in the absence of excipients. On contact with the antisolvent, indomethacin immediately precipitated, producing a milky suspension. The resulting suspension was maintained at a constant temperature of 25 °C under rapid agitation (1200 rpm) for 48 h, which was the time interval required for the solid-state form transformation from the initial α (metastable) to the γ form (stable). The seeds were analyzed by a Mastersizer 3000 (for method description, please see below). Powder X-ray diffraction (PXRD) analysis confirmed that the γ form was obtained. Additionally, scanning electron microscopy (SEM) images were acquired to check the particle morphology.

##### 2.2.2.2 Effect of Excipients and the Seeding Process on the Antisolvent Process

Production of crystalline indomethacin suspensions was accomplished with water as an antisolvent and ethanol as a solvent. The experimental conditions were optimized by monitoring the effect of the S/AS ratio (1:20 and 1:10), aging time (up to 1 week), and the effect of seeding on the resulting size, polymorphic form, and crystal habit after precipitation. LAS precipitation was conducted at 25 °C using a temperature-controlled water bath Grant TFX200 (Grant Instruments Ltd., Shepreth, U.K.).

To achieve the stable solid-state form of indomethacin, LAS precipitation was performed with seeding in the absence of excipients, at a seed concentration range of 1–4% w/v of the total volume, equivalent to 0.01–0.04% w/v of the indomethacin crystallizing mass, respectively. Samples were analyzed at different time points (1–24 h), and seeding was performed before and after nucleation of the API.

**Figure 1:**
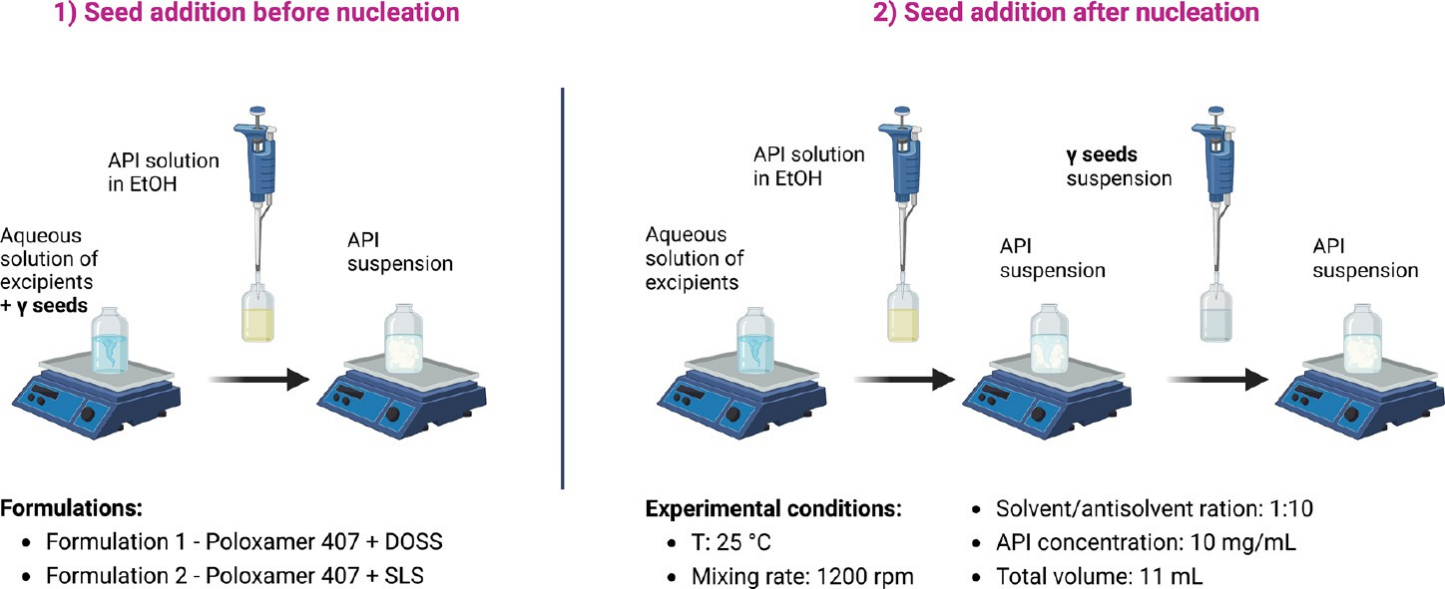
Schematic representation of the approaches used for seeding: (1) seed addition before nucleation, together with excipients and (2) seed addition after nucleation in the presence of excipients. The solid state of the seeds is the γ form, and seeds with a D50 of ∼17.1 ± 0.2 μm were used.

The impact of SLS, DOSS, and poloxamer 407 on the relative kinetics of nucleation, particle growth, and stabilization of the suspensions after formation of the particles was then investigated. The impact of the selected stabilizer, aging time, and the time of addition, concentration, and particle size of the seeds on the polymorphic form and the particle size of the API suspension were explored (Figure 1).

#### 2.2.3 Powder X-ray Diffraction

PXRD was used to identify the polymorphic form and to monitor the degree of crystallinity of the initial and processed samples. The particles in suspension were isolated through a simple Buchner filtration setup using a 0.22 μm pore size filter paper and dried in the fume hood overnight. Diffraction patterns were recorded using a PANalytical Empyrean (Malvern PANalytical Ltd., Malvern, U.K.) diffractometer in the reflection mode using Cu Kα radiation (λ = 1.54 Å) at 40 kV and 40 mA. Powder samples were prepared by adding a small amount of powder (filtered sample dried in the fume hood at room temperature) to zero background disks and scanning the samples in the angular range of 5° (2θ) to 40° (2θ) with 0.026° 2θ/min step size and 113 s per step, on a flat stage that was spinning at 4 rpm.

#### 2.2.4 Particle Size Distribution

The PSD of the indomethacin suspensions was determined using a Malvern Mastersizer 3000 (Malvern Panalytical Ltd., Malvern, U.K.), with water as the dispersion medium. An obscuration rate of 4–8%, a stirring speed of 1500 rpm, and a premeasurement delay of 10 s were used for each measurement. The refractive index was set at 1.68 and the absorption index at 0.01. The analysis was done using the General Purpose Model. Three measurements were taken per run, and the experiment was performed in triplicate. The average D10, D50, and D90 particle sizes refer to sizes where 10, 50, and 90% of the total volume of the material in the sample are contained, respectively, and the standard deviations were recorded for each sample.

#### 2.2.5 Scanning Electron Microscopy

The shape of the isolated crystals was characterized using a HITACHI SU-70 (Hitachi Inc., Japan) SEM instrument. The particles in the suspension were isolated through a simple Buchner filtration setup, using a 0.22 μm pore size, and dried in the fume hood overnight. To prepare the SEM sample, a small amount of the isolated particles was placed onto an adhesive carbon tape previously attached to a cylindrical aluminum 15 mm SEM stub. The samples were coated with gold using an Emitech K550 (Emitech, U.K.) sputter coater at 20 mA for 40 s. The particles were imaged at a voltage range of 5–10 kV. All SEM images shown in this work were fully representative of the entire sample analyzed in each case.

## 3 Results and Discussion

### 3.1 Preparation of Indomethacin Suspensions by Liquid Antisolvent Precipitation—without Seeding

The target particle size for an LAI suspension depends inherently on the solubility of the API and its desired dissolution rate at the site of administration, i.e., the duration of the pharmacological effect of the compound following administration of the LAI suspensions to the patient. For this study, the target particle size was defined as the range of 5–10 μm, which would allow for good injectability. When the LAS precipitation was performed in the absence of excipients, a range of particle sizes could be obtained by varying the temperature, S/AS ratio, stirring rate, and aging time (see Table S1). With ethanol as a solvent, at 5 °C with an S/AS ratio of 1:10, a stirring rate of 1200 rpm, and an aging time of 1–5 min, a higher and broader PSD than the target was obtained for the particles in the suspension. However, when a selection of these samples was examined by PXRD, they were found to be the metastable α form of indomethacin and not the desired stable γ form (Table S1). Furthermore, while maintaining the same experimental conditions, when the aging time was increased up to 30 min, the particle size grew spontaneously to sizes greater than 75 μm, and the polymorphic form obtained was still the α form (Table S1). However, at 25 °C when the aging time was 48 h, while maintaining the other parameters constant, the desired γ polymorphic form was achieved.

**Figure 2:**
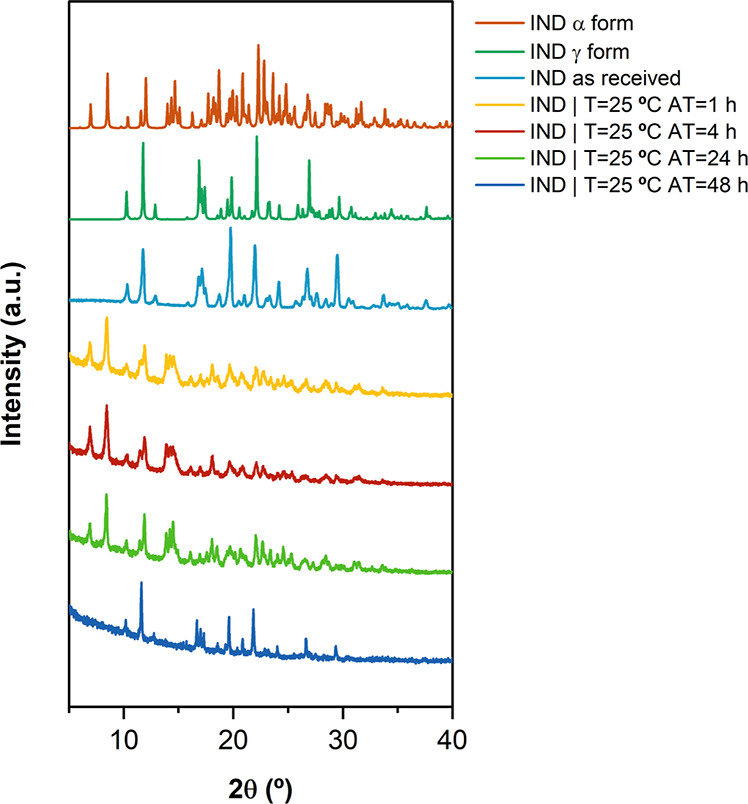
PXRD pattern of the indomethacin particles isolated from the suspension produced in the absence of excipients over time to determine the kinetics of the solid-state form transition from metastable (α) to stable (γ), in comparison with the product as received and the PXRD patterns sourced from the Cambridge database (INDMET02—α form; and INDMET03—γ form). The PXRD pattern acquired using Cu Kα radiation (λ = 1.54 Å) at 40 kV and 40 mA.

**Figure 3:**
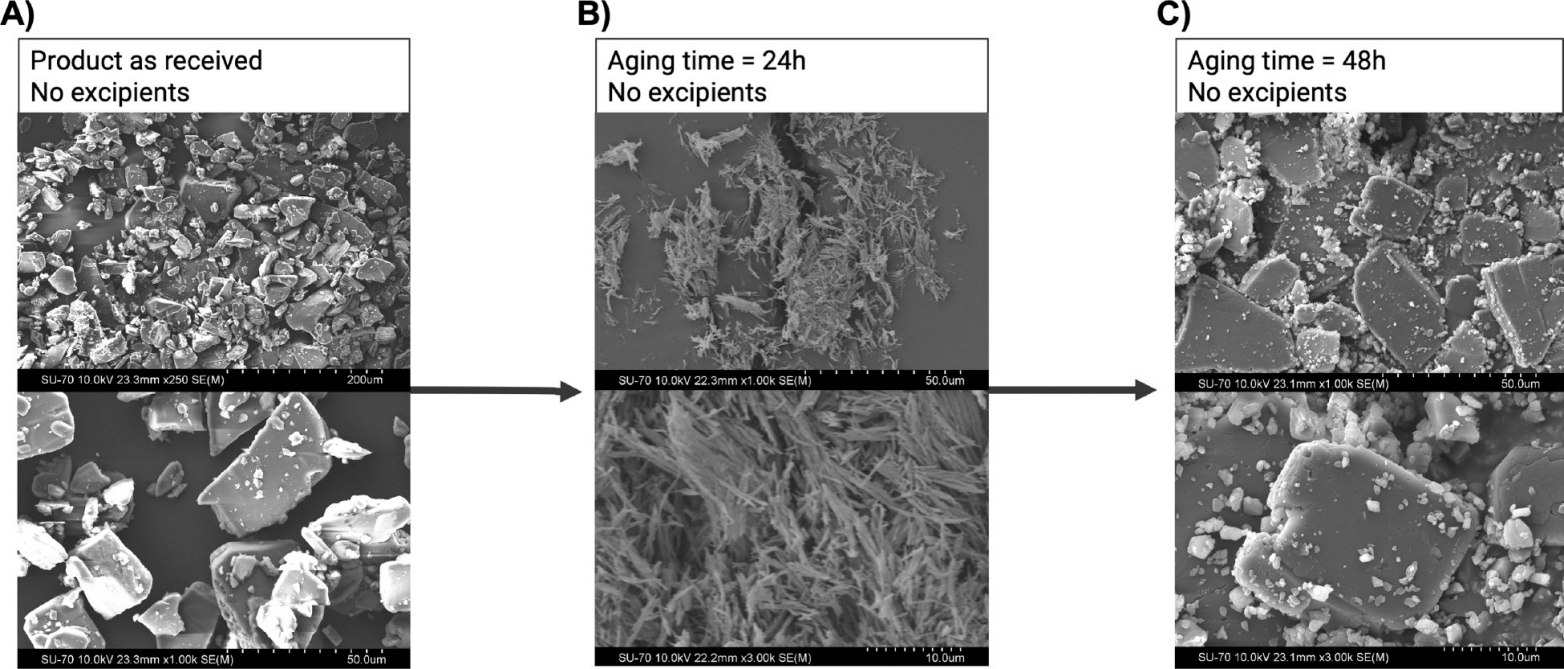
SEM images of indomethacin microparticles in the absence of excipients. (A) Indomethacin as received from the supplier; (B) indomethacin microparticles produced by LAS precipitation, after aging for 24 h—α form (needle shape); and (C) indomethacin microparticles produced by LAS precipitation, after aging for 48 h—γ form (rhombic plates).

Thus, at these conditions, i.e., a temperature of 25 °C, a stirring rate of 1200 rpm, an S/AS ratio of 1:10, an API concentration of 10 mg/mL, using ethanol as a solvent, and an aging time of 48 h, the kinetics of conversion of the α form to the γ form was monitored by PXRD and SEM as a function of time (Figures 2 and 3). It was observed that after 48 h, the transition from the metastable α form to the γ form was complete. These process parameters allowed the production of γ seeds with a D50 of 17.1 ± 0.23 μm that were used as seeds in subsequent experiments. Unfortunately, the target PSD, 5–10 μm, could not be obtained using these process parameters, although the stable form of the API was obtained.

**Figure 4:**
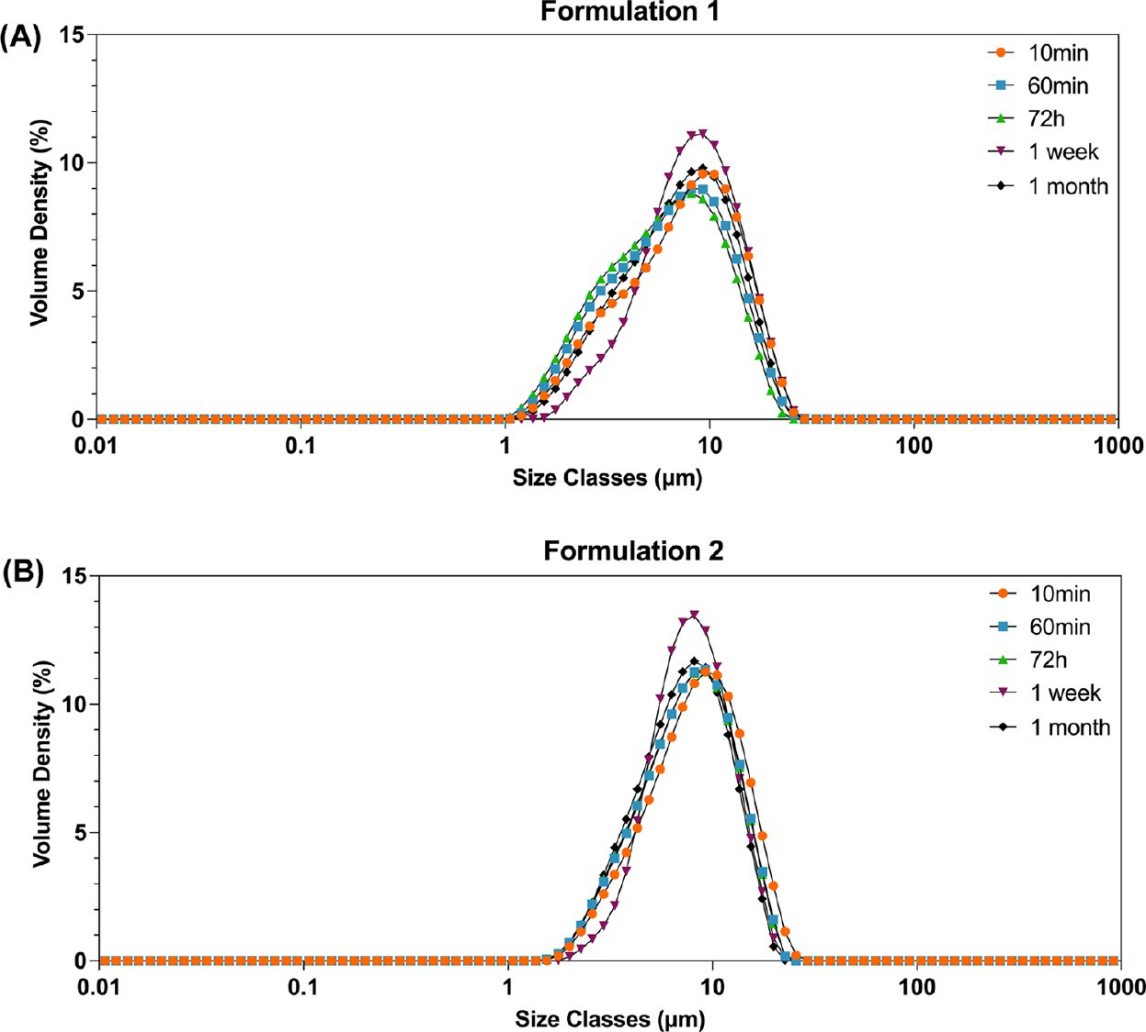
PSD over time for (A) formulation 1 (surfactant: DOSS 0.05% w/v; polymer: poloxamer 407 0.2% w/v) and (B) formulation 2 (surfactant: SLS 0.2% w/v; polymer: poloxamer 407 0.2% w/v). The data are presented for the aging times indicated.

To achieve the target PSD and the stable polymorphic form in the suspension, excipients were added to the formulation (Tables 3 and S2). Different modes of addition of surfactants and/or polymers were tested to better understand their role in the nucleation process and the stabilization that occurs afterward. Using the LAS precipitation approach with different excipients added to the aqueous phase, as described above (Section 2.2.2), two formulations generated indomethacin suspensions with D50 values within the target range (5–10 μm), see Table 4, although the full PSD was outside of the targeted range (Figure 4). The other formulations tested presented D50 values that were out of the target range and typically had a broader PSD (Table S2).

**Table 4:** Excipient Combinations and Respective PSDs of the Formulations from an Indomethacin Concentration of 10 mg/mL and an S/AS Ratio 1:10 at a Stirring Rate of 1200 rpm for Ethanol as a Solvent at 25 °C^a^

concepts	aging time	excipients	D10 (μm)	D50 (μm)	D90 (μm)
formulation 1	10 min	5 mL of poloxamer 407 0.2% w/v 5 mL of DOSS 0.05% w/v	2.77 ± 0.09	7.74 ± 0.45	15.50 ± 0.78
	72 h		2.54 ± 0.10	8.29 ± 0.64	14.00 ± 0.99
	1 week		3.87 ± 0.39	7.33 ± 0.38	15.50 ± 1.14
	1 month		2.92 ± 0.14	7.13 ± 0.28	14.50 ± 0.57
formulation 2	10 min	5 mL of poloxamer 407 0.2% w/v 5 mL of SLS 0.2% w/v	3.81 ± 0.03	8.46 ± 0.34	15.50 ± 0.90
	72 h		3.58 ± 0.03	7.86 ± 0.49	14.10 ± 0.23
	1 week		4.42 ± 0.29	7.36 ± 0.31	13.40 ± 0.97
	1 month		3.52 ± 0.13	8.49 ± 0.21	13.20 ± 0.54

a The excipients indicated were present at the time of addition of the drug solution to the antisolvent.

Based on these experiments, the most promising excipients for indomethacin suspension production by LAS precipitation were poloxamer 407 in combination with SLS or DOSS. For both formulations, the surfactant choice was not as critical as the polymer to obtain the targeted PSD (Tables 4 and S2). This might be due to the fact that the polymer plays a critical role in steric stabilization of the particles. PXRD analysis of the materials produced from the indomethacin suspensions, kept at 25 °C, was performed at different time points to monitor the kinetics of the polymorphic transformation.

**Figure 5:**
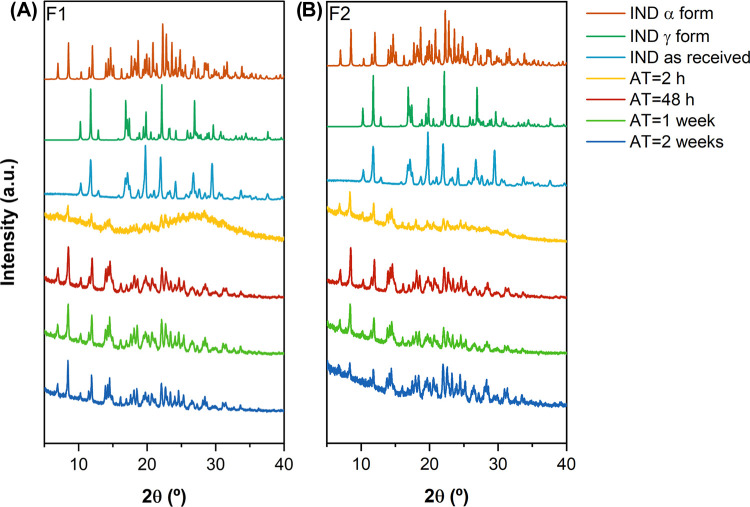
PXRD patterns of the different indomethacin samples for (A) formulation 1 (surfactant: DOSS 0.05% w/v; polymer: poloxamer 407 0.2% w/v) and (B) formulation 2 (surfactant: SLS. 0.2% w/v; polymer: poloxamer 407 0.2% w/v), in comparison with the product as received and the PXRD patterns sourced from the Cambridge database (INDMET02—α form and INDMET03—γ form). The PXRD pattern acquired using Cu Kα radiation (λ = 1.54 Å) at 40 kV and 40 mA.

As shown in Figure 5, the precipitated microparticles had a different solid-state form than the starting material powder, with a diffraction pattern corresponding to the metastable α polymorph. The PXRD pattern of the γ form (as received and from CSD INDMET03) showed characteristic intensity diffraction peaks at 2θ = 10.2, 11.8, 17.0, 19.9, and 21.9°; the α (CSD INDMET02) form presents at 2θ = 7.0, 8.5, 11.6, 12.0, and 14.0°.^61^ No polymorphic change was observed within a period of 2 weeks for either of the formulations.

**Figure 6:**
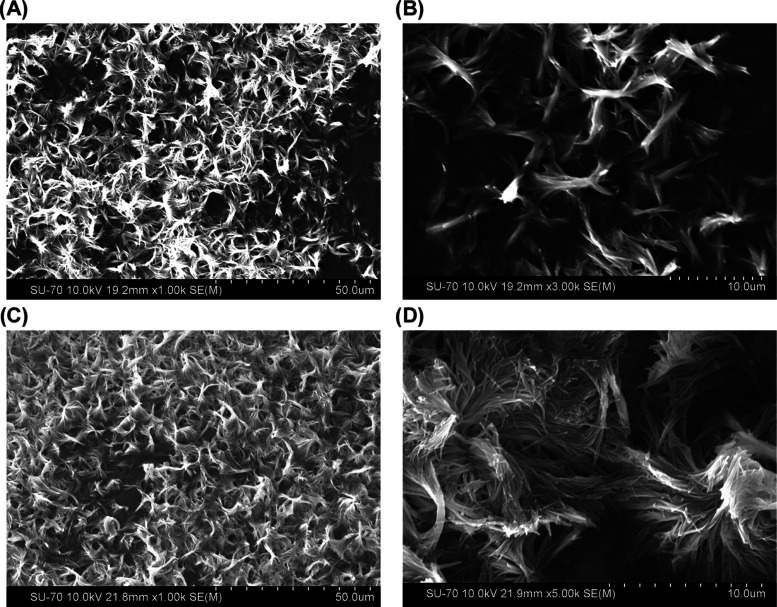
SEM images of indomethacin microparticles produced by LAS precipitation after aging for 72 h. (A, B) Formulation 1 (surfactant: DOSS 0.05% w/v; polymer: poloxamer 407 0.2% w/v) ×1000 and ×3000 and (C, D) formulation 2 (surfactant: SLS 0.2% w/v; polymer: poloxamer 407 0.2% w/v) ×1000 and ×3000.

To confirm the information acquired through PXRD, SEM imaging of the particles was performed for the two formulations. The resulting images are presented in Figure 6.

Both formulations were made of twisted and intertwined needle-shaped particles, typical of the α polymorph, as confirmed by the PXRD patterns obtained for these formulations.^62^ The images at lower magnifications show the overall structure of the sample.

Although excipients played a critical role in achieving the target PSD, their addition hindered the transition from the metastable form (α) to the stable form (γ) of the API particles (Figures 5 and 6).^43^

Slavin et al. investigated the polymorphic nature of crystalline indomethacin grown from a wide range of solvents. It was reported that growth in most solvents at high supersaturations resulted in the α form, whereas at low supersaturations, the γ form was commonly produced when pure solvents were used.^63^ Legendre and Feutelais reported that the metastable form of indomethacin (α) at room temperature and atmospheric pressure could lead to formulation difficulties, particularly in suspensions. The differences and relationship between the stable and metastable forms described in this work were thus of primary importance from a pharmaceutical point of view.^64^ It became clear for our process that the temperature (25 °C), solvent (ethanol), and the high supersaturation of the system, required for obtaining the target particle size distribution, favored the α form. The impact of excipients on the PSD and stability of indomethacin suspensions, although not specifically for LAS precipitation, has previously been reported. Ferrar et al. performed a high throughput screening study using 28 excipients and combinations thereof to predict the most promising combinations to produce stable suspensions of indomethacin.^18^ The suspensions were made using a top-down approach, wet milling process, utilizing a resonant acoustic mixer.^18^ It was observed that SLS, polysorbate 80, and d-α-tocopheryl polyethylene glycol 1000 succinate (TPGS 1000) combined with PEG of lower molecular weight (<4000) and poloxamers of higher molecular weights were advised for the production of stable indomethacin suspensions.^18^ These results lead to the hypothesis that indomethacin needs a stabilizing excipient that is amphiphilic, but that also contains a sufficiently long hydrophobic chain. This correlated with the optimal polymer, poloxamer 407, from our study, but no data on the impact of excipient on the resulting polymorphic form have been reported in the literature.

As changing the parameters further (e.g., temperature, API concentration, solvent, excipients, and aging time) did not result in the transformation of the solid-state form of indomethacin from the α form (metastable) to the γ form (stable) even over 2 weeks (data not shown), a seeding approach was investigated.

### 3.2 Preparation of Indomethacin Suspensions by Liquid Antisolvent Precipitation—with Seeding

In the absence of excipients, it took 48 h to obtain a suspension containing the γ form of indomethacin. However, within this time period, the particle size grew outside of the target range. In the presence of poloxamer with either DOSS or SLS, the particle size remained within the target range, but the metastable form (α) persisted. Therefore, seeding was introduced to speed up the polymorphic transformation to the γ form in the presence of excipients, since it is known that seeds facilitate transformation of metastable forms to stable polymorphic forms.^46,49,65^

First, the impact of seeding on the rate of transformation in the absence of excipients was investigated. Therefore, keeping the same conditions as the LAS precipitation experiment described in Section 3.1, i.e., a temperature of 25 °C, an S/AS ratio of 1:10, a stirring rate of 1200 rpm, and a seed concentration of 2% w/v, the stable polymorphic form (γ form) was obtained after 24 h, half of the time required in the absence of seeds (Figure S1A). However, again it was observed that the PSD of the suspension seeds produced in the absence of excipients was not stable over time (Figure S1B).

The rate of solid-state transformation of the metastable to the stable polymorphic form depends on the solubility difference between the two forms.^66^ This has been suggested to be influenced directly by the tendency of the stable form to nucleate, the dissolution kinetics of the metastable form phase, the growth of the stable form, and the diffusion of mass between the two phases.^65,67,68^ Additionally, it has been shown in the work of Maher et al. that the low solubility of the metastable form in the solvent retards the nucleation and growth of the stable form.^69^ Hence, a similar mechanism may have occurred in the present study, due to the low solubility of the metastable form in the S/AS mixture, explaining the slow transformation rate into the stable form. Thus, considering that seeding leads to a faster solid-state form transformation, any change in the surface properties of the nucleated metastable form, such as surface adsorption of excipients, may affect this process.

To obtain the same target size as before, the seeding approach was tested in the presence of the excipients of the two successful formulations (Figure 1), and a range of variables were investigated including the concentration of the seeds, the PSD of the seeds, aging time, and time of seeding (i.e., before nucleation (SB) or after nucleation (SA)). Seeding before and after nucleation was done to investigate if the seeds needed to be present during nucleation to drive the transformation to the stable form (Table 5).

**Table 5:** Impact of the Percentage w/v of Indomethacin Seeds on the Resulting Polymorphic Form of Indomethacin in Formulation 1 (Surfactant: DOSS 0.05% w/v; Polymer: Poloxamer 407 0.2% w/v) and Formulation 2 (Surfactant: SLS 0.2% w/v; Polymer: Poloxamer 407 0.2% w/v) at 4 and 24 h^a^

	formulation 1 | SB			formulation 1 | SA		
	form by PXRD			form by PXRD		
aging time (h)	1% w/v	2% w/v	4% w/v	1% w/v	2% w/v	4% w/v
4	α	α	α	α	α	α
24	α	α	α	α	α	α

	formulation 2 | SB			formulation 2 | SA		
	form by PXRD			form by PXRD		
aging time (h)	1% w/v	2% w/v	4% w/v	1% w/v	2% w/v	4% w/v
4	γ	γ	γ	α	α	α
24	γ	γ	γ	α	γ	α

a SB, seed addition before nucleation; SA, seed addition after nucleation.

It was possible to consistently obtain the stable solid form, the γ form, after 4 h when the seeds of the desired polymorphic form were added to the antisolvent (at all concentrations tested) before nucleation with formulation 2, i.e., the formulation containing poloxamer 407 and SLS. Additionally, for formulation 2, it was also verified that the solid-state transformation to the γ form occurred for a seed concentration of 2% w/v added after nucleation (SA), after an aging time of 24 h (Table 4 and Figures S2 and S3). The transformation was not observed at 1 or 4% w/v concentrations of seeds when they were added after nucleation. Figure S4 presents the PXRD patterns of indomethacin isolated from formulations 1 and 2, indicating that the γ form was consistently obtained when SLS was used as the surfactant and when the addition of γ seeds to the antisolvent took place before nucleation.

**Figure 7:**
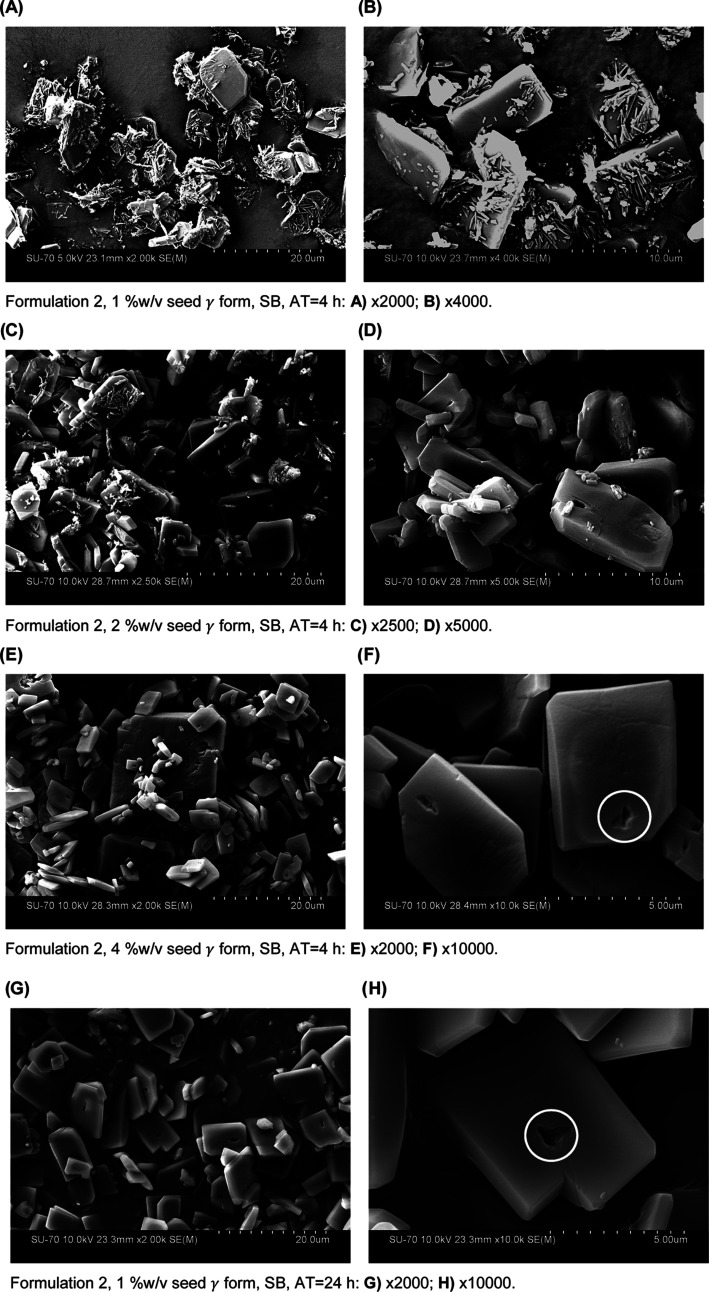

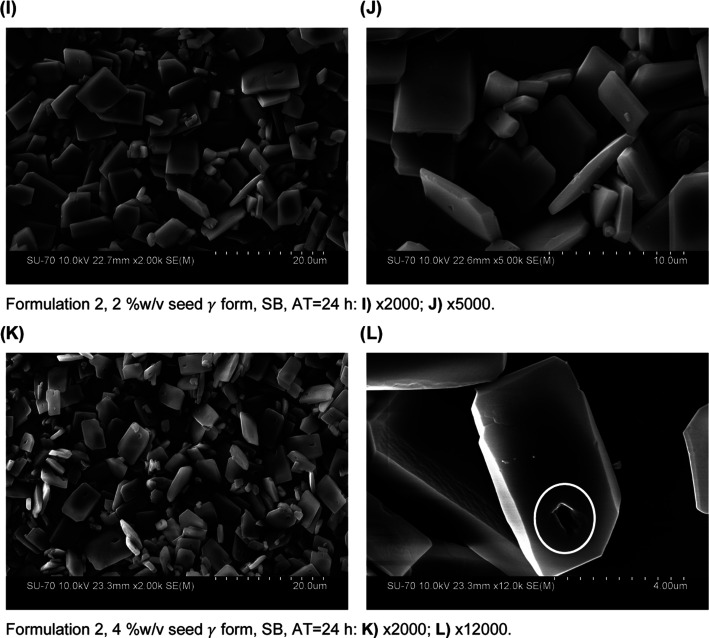
SEM images of indomethacin microparticles produced by LAS precipitation, formulation 2 (surfactant: SLS 0.2% w/v; polymer: poloxamer 407 0.2% w/v). Legend: white circles—cavities seen in crystals after the solid-state form transformation.

Figure 7 presents SEM images of indomethacin isolated from formulation 2, showing that the stable form, γ, was consistently obtained when SLS was used as the surfactant and when the addition of the γ seeds took place before nucleation.

The images presented in Figure 7 correspond to formulation 2, the only tested formulation where it was possible to observe solid-state transformation, from the metastable to the stable form. The main difference between formulations 1 and 2 was the surfactant used, meaning that SLS, i.e., the surfactant used in formulation 2, allowed for the solid-state transition from the metastable form (α form) to the stable form (γ form) of indomethacin, while DOSS, the surfactant used in formulation 1, did not. As shown in Figure 7A–D, in experiments with seeds added before nucleation at 4 h (as described in Section 3.2), needle-shaped structures (α form) were observed, together with rhombic plates (γ form), which showed that the transition from α to γ was still happening, i.e., the formation of the α form and its conversion into the γ form assisted by the seed surface. In Figure 7E–L, i.e., experiments with seeds added to the antisolvent before nucleation at 24 h (as described in Section 3.2), the rhombic plates that are characteristic of the indomethacin γ form could clearly be observed, with little to no needles (α form) observed.^62^ Additionally, the picture shown in the right column, taken at a higher magnification, reveals more details of the particles’ habits, featuring the presence of a hole in each crystal (white circles in Figure 7). It may be hypothesized that the formation of this hole might be due to the transition mechanism from the metastable, α form, to the stable form, γ form, of indomethacin. Further investigation was considered out of the scope of this study. With increasing seed concentration, the rate of transformation from the metastable to the stable form increased (Figure 7A–F). Also, 24 h after nucleation, when seeding happened before nucleation, no differences were observed at different seed concentrations (Figure 7G–L), indicating that the transformation into the γ form was complete in this time.

**Figure 8:**
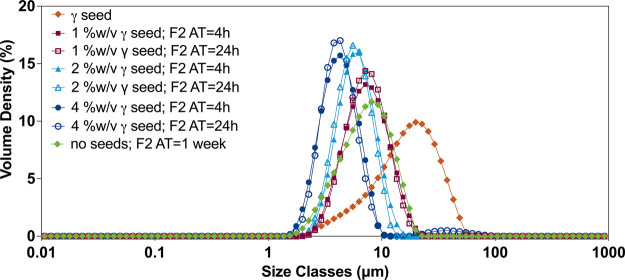
PSD of formulation 2 of indomethacin and γ seeds. Comparison of the PSD of the formulation with and without the seeding approach at 4 and 24 h for the three seed concentrations tested. Formulation 2 (surfactant: SLS 0.2% w/v; polymer: poloxamer 407 0.2% w/v).

The particle size distributions obtained for formulation 2 with different concentrations of seeds at 4 and 24 h are presented in Figure 8.

Formulation 2, independent of the seed concentration, had a D50 value between 5 and 10 μm with a narrow distribution (Table S3), i.e., within the target defined in this study, with the stable γ form after 4 h. For seed concentrations of 2 and 4% w/v, the PSD obtained was within the target range of this study (5–10 μm), after aging times of 4 and 24 h.

The γ seeds used for the seeding had a size of ∼17.1 ± 0.2 μm (D50) and a broad PSD, as presented in Figure 8 (⧫), while the D50 of the final suspension was 5–10 μm with a narrow size distribution (Table S3). When the PSD obtained was compared with the same formulation without the seeding, where indomethacin remained in the metastable α form, a similar PSD was obtained, Figure 8 (⧫). Thus, although seeding was critical to obtain the stable solid-state form (γ), it was not a critical process parameter to obtain the target PSD of the indomethacin suspension.

A narrower particle size distribution was obtained when seeding was done before the nucleation (SB) than after the nucleation (SA) (Figure S5). This difference could potentially affect the dissolution profile and stability of the final suspension, but additional studies would need to be conducted to confirm this result. When experiments were performed with seeds having a D50 value greater than 30 μm, the solid-state polymorphic transformation ceased to happen, indicating that the size of the initial seeds is critical for the final solid-state form of the suspended particles (Table S4 and Figure S6).

**Figure 9:**
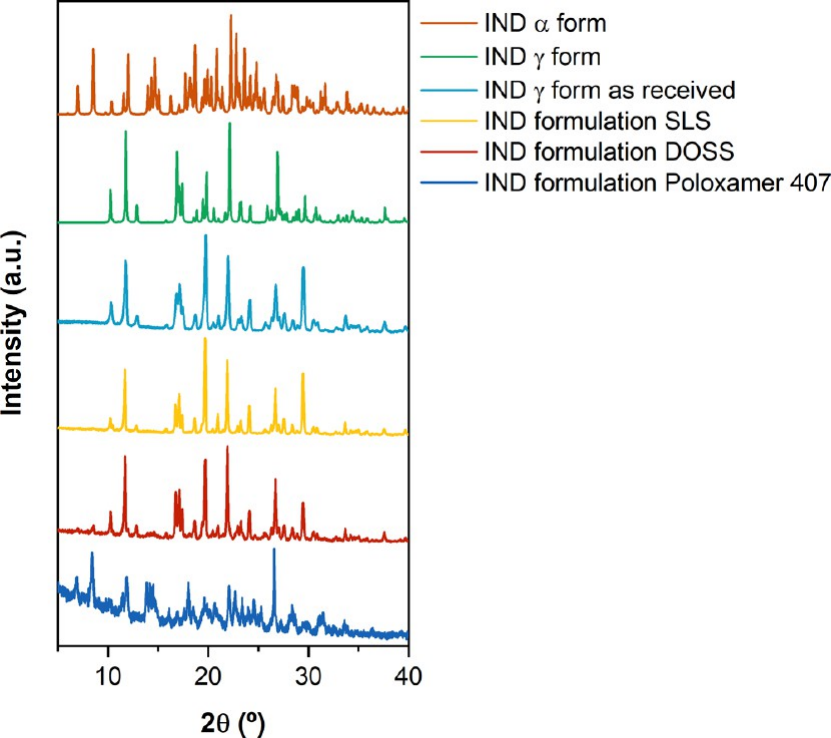
PXRD patterns of the different indomethacin samples for formulation with SLS 0.2% w/v; DOSS 0.05% w/v; and poloxamer 407 0.2% w/v, in comparison with the product as received and the PXRD patterns sourced from the Cambridge database (INDMET02—α form; and INDMET03—γ form). The PXRD pattern acquired using Cu Kα radiation (λ = 1.54 Å) at 40 kV and 40 mA.

To better understand which excipients (i.e., DOSS, SLS, or poloxamer 407) were affecting the solid-state transformation of indomethacin, a suspension of indomethacin particles was generated via the LAS process in the presence of the individual excipients without seeds and monitored over 48 h (Figure 9).

Complete transformation into the γ form was observed when SLS was the only excipient used (Figure 9). With DOSS alone, there was a partial transformation after 48 h, and for poloxamer 407, no transformation was observed, with the suspended particles remaining as the α form. Note the target particle size distribution could not be achieved when only single additives were present (data not shown).

**Figure 10:**
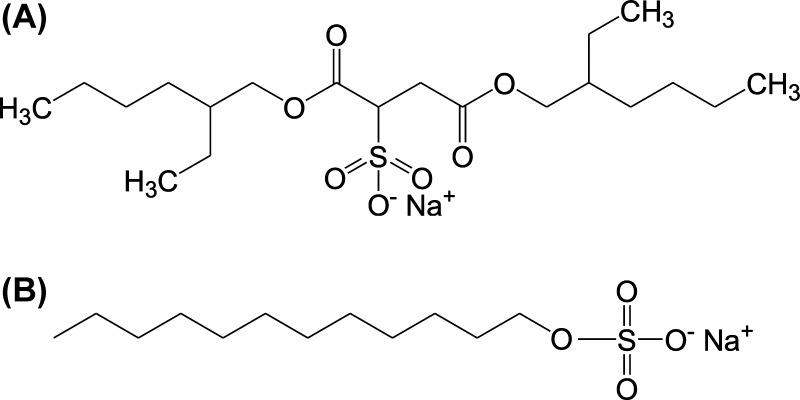
Chemical structures of (A) SLS and (B) DOSS.

The only difference between formulations 1 and 2 was the chosen surfactant, formulation 1 had DOSS and formulation 2 had SLS. Nonetheless, as observed in Figure 9, the presence of poloxamer inhibits completely the polymorphic transformation of indomethacin. DOSS has a longer chain and is branched when compared to SLS (Figure 10), which may result in stronger adsorption of DOSS onto the precipitated particles’ surface and/or stronger interactions between DOSS and indomethacin molecules in solution. We hypothesize that the combination of poloxamer 407 and DOSS thus may inhibit the interaction of indomethacin in the molecular form with the surface of the added seeds. In general, when the seeds were added before nucleation, the solid-state transition occurred consistently (from the α form to the γ form; Table 4 and Figures S4 and S7). It may be hypothesized that when the seeds were added after nucleation, the excipients had already surrounded and adsorbed onto the precipitated particles’ surface or were interacting with dissolved indomethacin, preventing the molecules of API from interacting with the surface of the seeds and, consequently, preventing the solid-state form transition from occurring (Figures S2 and S3).

When experiments were performed with seeds having a mean particle size higher than 30 μm, no solid-state transition was observed and much larger particles with a broad PSD were obtained. This observation also supported our hypothesis for explaining the effect of seeds on the polymorphic form transformation, since seed particle size, and thus available surface area, has a large influence on the rate of transformation (Table S4 and Figure S6). This observation could indicate that the size of the initial seeds was critical for obtaining both the aimed polymorphic form and the target particle size. This result was in accordance with the conclusion of He et al., who stated the importance of the size distribution of seeds on the size of the resulting crystals and crystal size distribution.^70^ This may be explained by the active surface area of the seed, which impacts the available surface area for crystal growth. Furthermore, a combination of factors can be critical during seeding for controlling secondary nucleation and crystal growth: size distribution and seed surface, and seed density or loading amount.^56,71,72^

## 4 Conclusions

Seeds of the stable polymorphic form of indomethacin drove the transition from the nucleated metastable form to the stable form during LAS precipitation. The rate of this transition was influenced by the presence of excipients, which were necessary to obtain the target particle size distribution. The present study showed that (1) excipients played a crucial role during nucleation and for long-term stabilization of the drug particle size distribution; (2) the solid-state form transformation rate from the metastable form (α) to the stable form (γ) could be expedited in the presence of seeds of the stable form; (3) excipients influenced the polymorphic form transition both in the absence and presence of seeds; (4) the size of the seeds was critical for defining the final solid-state form; and (5) the time point of seed addition was critical to obtain the aimed solid-state form. In summary, this alternative, energy-efficient bottom-up method to produce drug suspensions with a reduced risk of contamination from milling equipment and fewer processing steps may prove to be comparable in terms of stability and PSD to the current industrially accepted top-down approaches.

## Supporting Information Available

Additional experimental details on the screening of excipients; and complementary characterization of indomethacin suspensions: PXRD and PSD graphs, SEM pictures, and PSD (D10, D50, D90) (PDF)
