# Supplementary material for: Impact of Excipients and Seeding on the Solid-State Form Transformation of Indomethacin during Liquid Antisolvent Precipitation
Source: Cryst Growth Des. 2022 Sep 9;22(10):6056–69. doi: 10.1021/acs.cgd.2c00678 (PMC9542716; doi:10.1021/acs.cgd.2c00678)
Supplement: Supplementary file 1 — cg2c00678_si_001.pdf [file cg2c00678_si_001.pdf]

IMPACT OF EXCIPIENTS AND SEEDING ON THE SOLID-STATE FORM TRANSFORMATION OF INDOMETHACIN DURING LIQUID ANTISOLVENT PRECIPITATION

Mariana Hugo Silva[1,2], Ajay Kumar[2], Benjamin K. Hodnett[2], Lidia Tajber[3], René Holm[4], Sarah P. Hudson[2,*]

Supporting Information

[1] Pharmaceutical Product Development and Supply, Janssen Research and Development, division of Janssen Pharmaceutica NV, Beerse, Belgium

[2] Department of Chemical Sciences, SSPC the Science Foundation Ireland Research Centre for Pharmaceuticals, Bernal Institute, University of Limerick, Castletroy, Co. Limerick, Ireland

[3] School of Pharmacy and Pharmaceutical Sciences and the Science Foundation Ireland Research Centre for Pharmaceuticals (SSPC), Trinity College Dublin, College Green, Dublin 2, Ireland.

[4] Department of Physics, Chemistry and Pharmacy, University of Southern Denmark, Odense, Denmark

*Corresponding Author:

Sarah P Hudson, Department of Chemical Sciences, SSPC the Science Foundation Ireland Research Centre for Pharmaceuticals, Bernal Institute, University of Limerick, Castletroy, Co. Limerick, Ireland, e-Mail: sarah.hudson@ul.ie; Tel.: +353 87 634 6006

Supporting Information

Screening of processing conditions for LAS precipitation

1. Without excipients

Table S1 - *Summary of LAS process parameters tested for indomethacin in the absence of excipients, and respective D50 of the particles produced in suspension.*

API concentration	Solvent	Solvent/antisolvent ratio	Stirring rate	Temperature	Aging time	D50 (um)
50 mg/ml	ethanol	1:10	1200 rpm	5	1 min	32.6 ± 1.11
50 mg/ml	ethanol	1:10	1200 rpm	5	1 min	49.7 ± 0.707
25 mg/ml	ethanol	1:10	1200 rpm	5	1 min	24.6 ± 1.22
10 mg/ml	ethanol	1:10	1200 rpm	5	1 min	0.141 ± 0.012
10 mg/ml	ethanol	1:10	1200 rpm	5	5 min	0.109 ± 0.007
50 mg/ml	ethanol	1:10	500 rpm	5	1 min	28.8 ± 0.624
50 mg/ml	ethanol	1:20	1200 rpm	5	1 min	27.9 ± 0.553
25 mg/ml	ethanol	1:20	1200 rpm	5	1 min	24.6 ± 0.702
10 mg/ml	ethanol	1:20	1200 rpm	5	1 min	28.8 ± 1.27
10 mg/ml	ethanol	1:20	1200 rpm	5	10 min	541 ± 854
10 mg/ml	ethanol	1:20	1200 rpm	5	5 min	39.3 ± 1.61
10 mg/ml	ethanol	1:10	1200 rpm	5	5 min	0.111 ± 0.017
10 mg/ml	ethanol	1:10	1200 rpm	5	10 min	75.5 ± 6.07
10 mg/ml	ethanol	1:10	1200 rpm	5	30 min	99.7 ± 6.28

2. With excipients

To achieve the right PSD with the stable polymorphic form excipients were then introduced to the experimental design.

Table S2 - *Summary of LAS process parameters tested for indomethacin at 25 °C with AS/S ratio equal to 1:10, with ethanol as solvent.*

Aging time	Excipients	D10 (um)	D50 (um)	D90 (um)
10min	Pol 407 1%w/v	18.7 ± 6.33	257 ± 110	498 ± 658
10min	PVP K30 1%w/v	37.9 ± 17.3	92.5 ± 21.1	148 ± 25.1
10min	DOSS 0.1%w/v	39.8 ± 4.74	90.7 ± 17.6	174 ± 45.0
10min	HPMC 0.1%w/v	42.6 ± 3.02	87.7 ± 8.26	153 ± 6.86
1 min	DOSS 0.1%w/v + PVP K30 1%w/v	0.022 ± 0.001	0.076 ± 0.009	111 ± 9.22
30min		0.022 ± 0.001	0.080 ± 0.007	876 ± 708
60min		0.021 ± 0.001	0.066 ± 0.006	2.73 ± 2.12
1 min		0.022 ± 0.001	0.078 ± 0.007	668 ± 462
60min		0.021 ± 0.002	0.071 ± 0.016	164 ± 9.10
1min		0.021 ± 0.002	0.066 ± 0.002	14.7 ± 25.0
1min		0.022 ± 0.001	0.062 ± 0.006	0.187 ± 0.030
1min		0.029 ± 0.001	7.05 ± 0.789	116 ± 17.6
1min	PVP K30 1%w/v	0.026 ± 0.002	0.165 ± 0.069	167 ± 9.02
30min		39.4 ± 37.1	60.4 ± 56.5	600 ± 805
10 min		0.022 ± 0.001	0.078 ± 0.016	678 ± 1040
1min	DOSS 0.1%w/v + Pol 407 1%w/v	8.42 ± 7.28	42.8 ± 11.4	498 ± 568
1min		8.93 ± 7.68	47.7 ± 7.61	171 ± 24.5
30min		0.041 ± 0.001	25.3 ± 2.40	118 ± 2.80

10min		0.022 ± 0.001	0.074 ± 0.003	5.30 ± 8.65
1min	SDS 0.1%w/v + Pol 407 1%w/v	9.89 ± 0.45	23.1 ± 0.066	47.0 ± 1.67
10min		11.1 ± 0.913	20.6 ± 1.63	34.6 ± 2.87

Seeding Approach

a) Seeding without excipients

A)

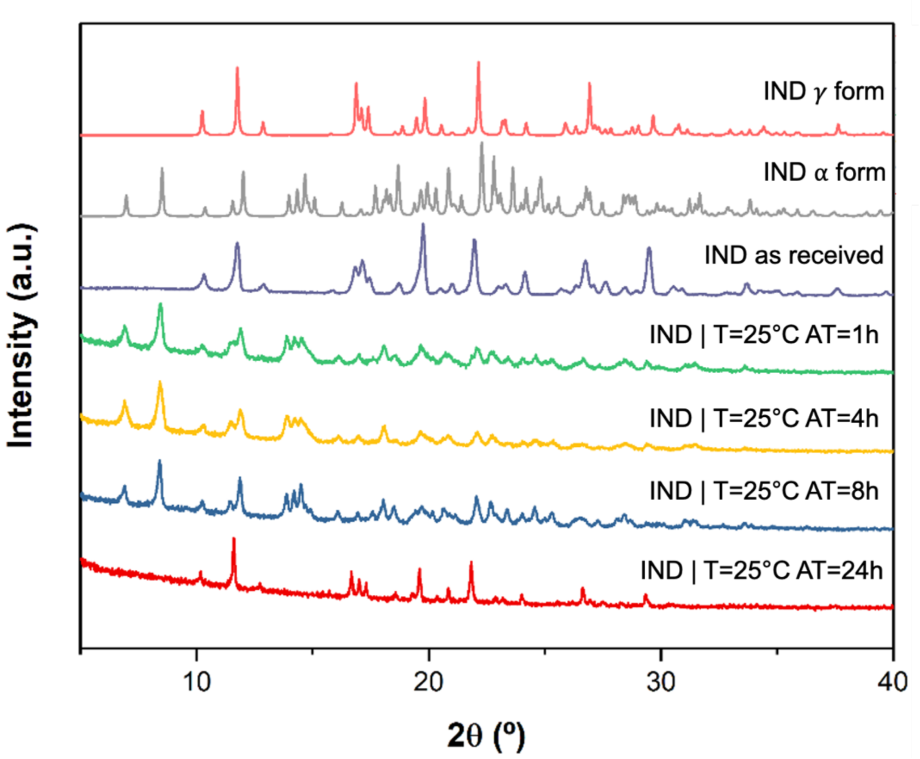

B)

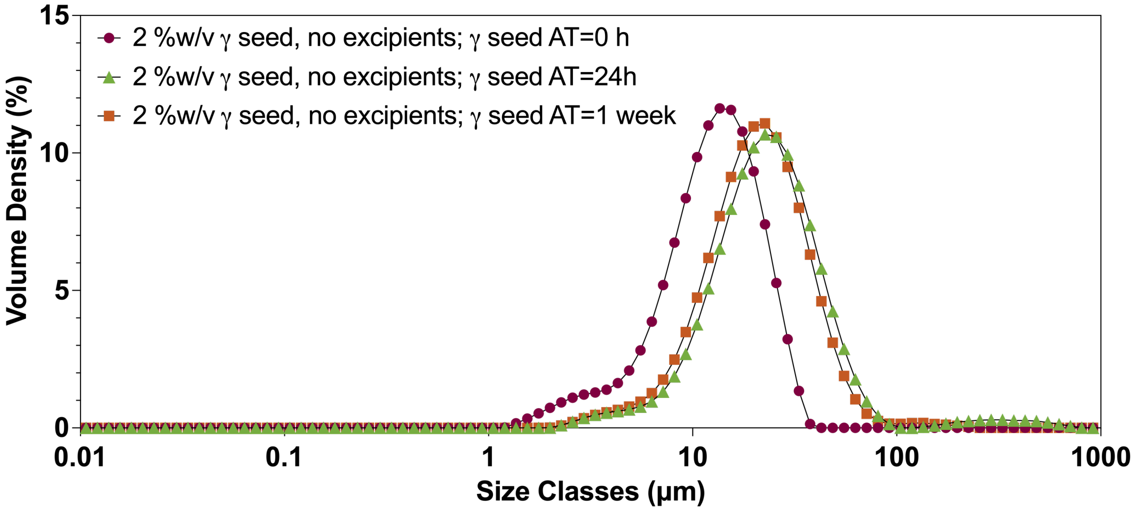

Figure S1 *– A) PXRD pattern of the indomethacin particles isolated from suspension produced with the seeding approach over time to determine the kinetics of the solid-state form transition from metastable (α) to stable (γ). In comparison with the product as received and the PXRD patterns from Cambridge database (INDMET02 – α form; and INDMET03 – γ form). PXRD pattern acquired using Cu-Kα radiation (λ = 1.54 Å) at 40 kV and 40 mA. B) Comparison of the PSD of the γ seed with the seeding approach in the absence of excipients at 0, 24 h and 1 week.*

b) Seeding before nucleation

Table S3 - *Impact of percentage w/v of indomethacin seed on PSD (D10 and D90) at 4 and 24 hours for SB – seed addition before nucleation.*

	**Formulation 2 | SB**					
	**D10 (µm)**			**D90 (µm)**		
**Time point**	**1%w/v**	**2%w/v**	**4%w/v**	**1%w/v**	**2%w/v**	**4%w/v**
4h	4.13 ± 0.13	3.69 ± 0.03	2.61 ± 0.16	12.9 ± 0.54	9.42 ± 0.26	6.64 ± 0.12
24h	4.23 ± 0.01	3.55 ± 0.06	2.73 ± 0.07	12.1 ± 0.34	8.70 ± 0.27	6.70 ± 0.31

c) Seeding after nucleation

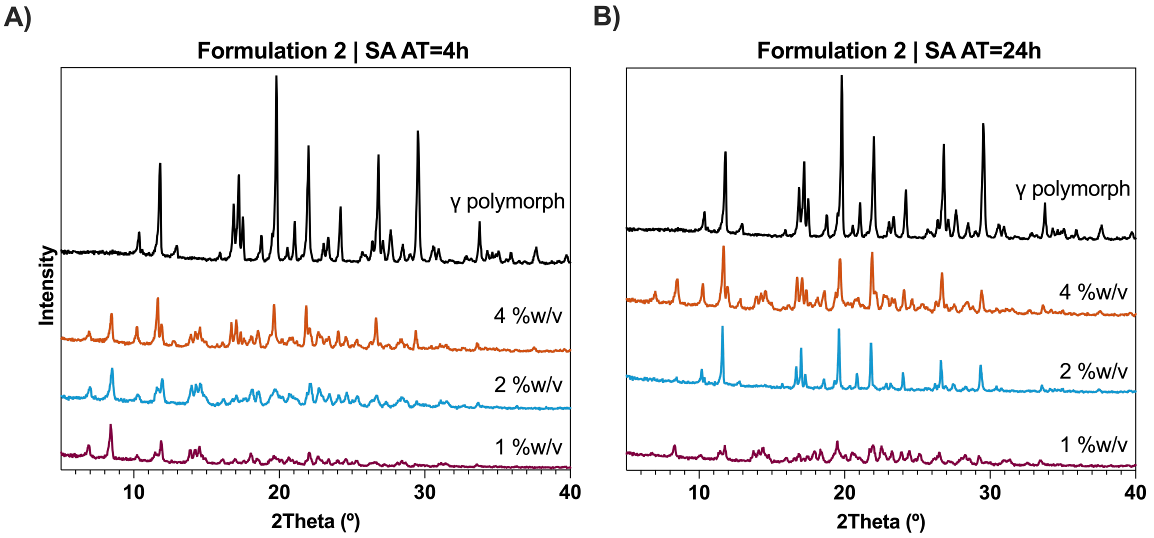

Figure S2 - *PXRD pattern of the formulation 2 with seeding after (SA) nucleation at (A) 4 hours and (B) 24 hours. PXRD pattern acquired using Cu-Kα radiation (λ = 1.54 Å) at 40 kV and 40 mA.*

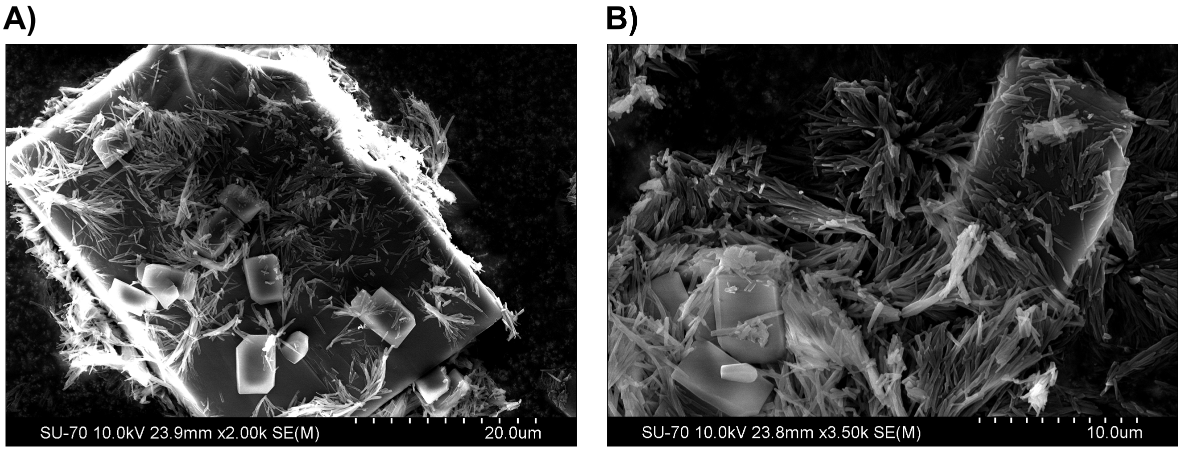

Figure S3 - *SEM images of indomethacin microparticles particles produced by LAS precipitation. Formulation 2, 2 %w/v seed γ form, SA, AT=24h: A) x2000; B) x3500.*

As shown in **Figure S3,** i.e. experiments with seed added after nucleation, needle structures (α form) were observed together with rhombic plates (*γ* form), which shows the transition phase, i.e the conversion into the stable form is occurring. A possible explanation for the images captured may be that the later addition of seed slowed and/or interfered with the solid-state form transformation, leading to an incomplete transition to the stable solid-state form (*γ* form).

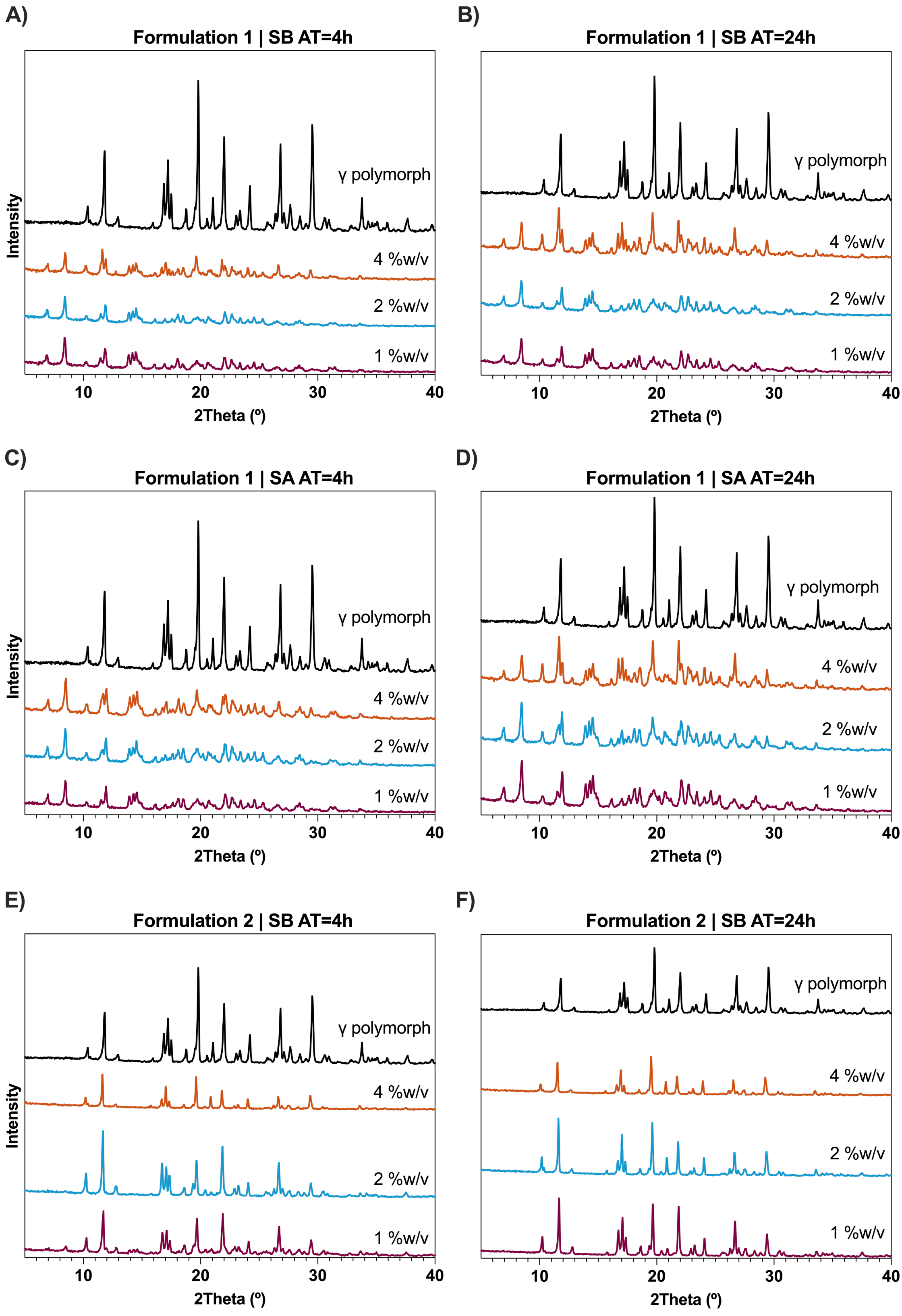

***Figure S4** - PXRD pattern of the A-D) formulations 1 (surfactant: DOSS 0.05 %w/v; polymer: poloxamer 407 0.2 %w/v); and E-F) formulation 2 (surfactant: SLS 0.2 %w/v; polymer: poloxamer 407 0.2 %w/v) with seeding*

before (SB) and after (SA) nucleation at 4 hours and 24 hours. PXRD pattern acquired using Cu-Kα radiation (λ = 1.54 Å) at 40 kV and 40 mA.

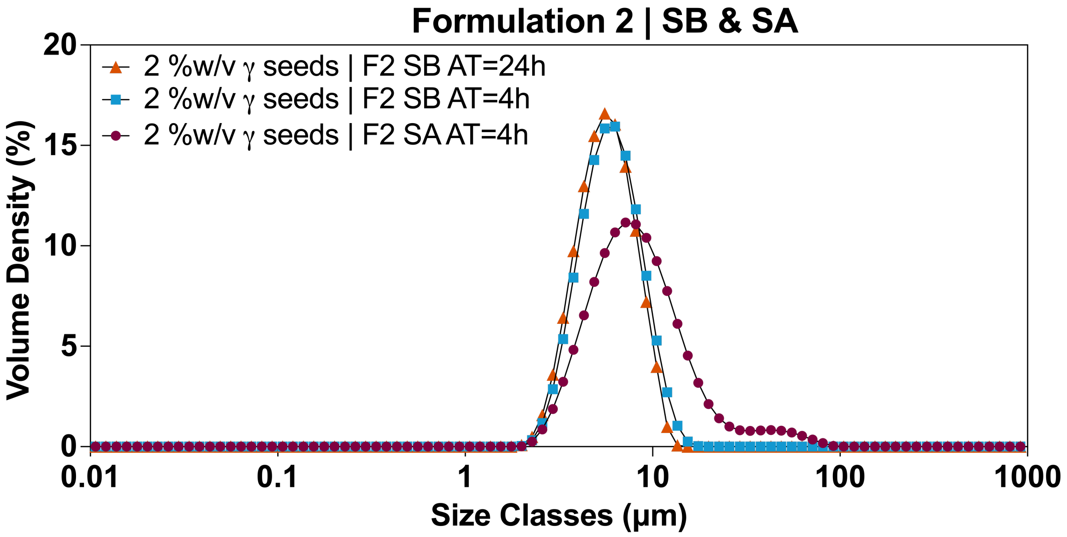

Figure S5 *- PSD of the formulation 2 (containing the γ form), at 4h and 24h, for the seed concentration of 2%w/v.*

As shown in **Figure S5,** a narrower particle size distribution was obtained when seeding was done before the nucleation (SB) than after the nucleation (SA). This difference could potentially affect the dissolution profile and stability of the final suspension, but additional studies would need to be conducted to make further conclusions.

d) Seeding experiments that resulted in no change in the solid-state form

Table S4 *- PSD of the seed used for LAS precipitation combined with seeding approach. Legend: BP – batch process.*

Sample ID	D10 (µm)	D50 (µm)	D90 (µm)
BP S1	20.7 ± 0.491	54.0 ± 0.762	103 ± 2.96
BP S2	11.8 ± 0.0396	31.5 ± 0.338	61.9 ± 1.44

A)

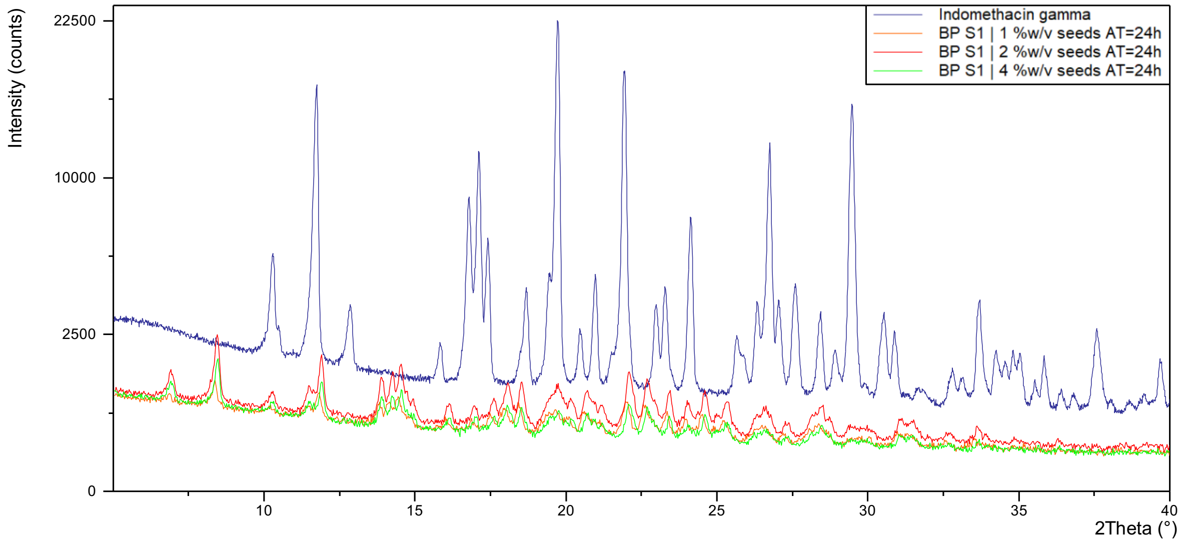

B)

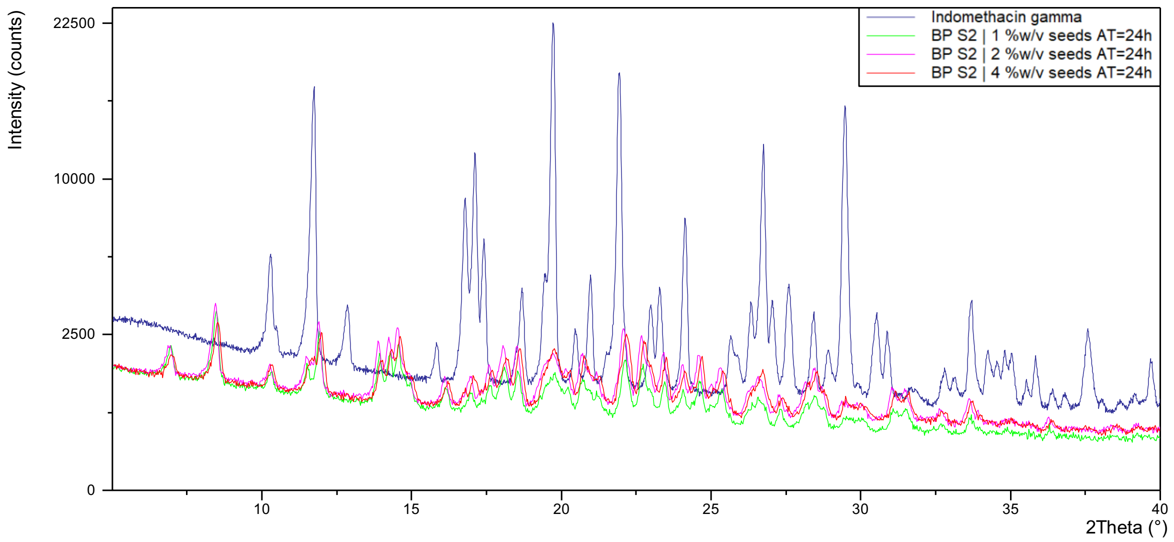

Figure S6 – *PXRD pattern of the suspensions generated by LAS precipitation combined with seeding by two different batches of seeds: A) BP S1; B) BP S2. PXRD pattern acquired using Cu-Kα radiation (λ = 1.54 Å) at 40 kV and 40 mA.*
